# Effectiveness of hysteroscopic resection of a uterine caesarean niche can be predicted: a prospective cohort study

**DOI:** 10.1038/s41598-020-74622-8

**Published:** 2020-10-15

**Authors:** Qian Zhu, Xiaoqing He, Ling Jiang, Guiling Liang, Chenfeng Zhu, Hongjie Pan, Jian Zhang, Judith Anna Huirne

**Affiliations:** 1 Department of Obstetrics and Gynecology, International Peace Maternity and Child Health Hospital, School of Medicine, Shanghai Jiaotong University, Shanghai, China; 2 Shanghai Key Laboratory Embryo Original Diseases, Shanghai, China; 3 Department of Radiology, International Peace Maternity and Child Health Hospital, School of Medicine, Shanghai Jiaotong University, Shanghai, China; 4 Department of Obstetrics and Gynecology, Sir Run Run Shaw Hospital, School of Medicine, Zhejiang University, Hangzhou, China; 5 Department of Obstetrics and Gynaecology, Amsterdam University Medical Centers, Amsterdam Reproduction and Development Research Institute, Amsterdam, The Netherlands

**Keywords:** Diseases, Urogenital reproductive disorders

## Abstract

This study aimed to develop and validate a model for the preoperative prediction of the effectiveness of hysteroscopic resection of a uterine cesarean niche in patients with postmenstrual spotting. The predictive model was developed in a primary prospective cohort consisting of 208 patients with niche treated by hysteroscopic resection. Multivariable logistic regression analysis was performed to develop the predictive model, which incorporated preoperative menstrual characteristics and magnetic resonance imaging (MRI) findings. Surgical efficacy was defined as a decrease in postmenstrual spotting duration of at least 3 days at the 3-month follow-up compared with baseline. The predictive model was presented with a nomogram, and the performance was assessed with respect to its calibration, discrimination, and clinical use. Internal validation was performed using tenfold cross-validation. The predictive factors in the final model were as follows: preoperative menstrual duration, thickness of the residual myometrium (TRM), length, TRM/thickness of the adjacent myometrium ratio, angle γ, area, and presence of a lateral branch of the niche. The model showed good performance in predicting the effectiveness of hysteroscopic niche resection. Incorporating the preoperative duration of the menstrual period and MRI findings of the niche into an easy-to-use nomogram facilitates the individualized prediction of the effectiveness of a hysteroscopic niche resection by 26 Fr resectoscope, but multicenter prospective studies are needed to validate it.

## Introduction

Cesarean scar defect (CSD) in the uterine cesarean scar, also called a niche^1^, is one of the long-term complications after cesarean section (CS). According to the international niche taskforce group, a niche was defined as an indentation of at least 2 mm on the side of the uterine cesarean scar^2^. A niche can be examined using transvaginal sonography (TVS), with or without saline or gel contrast, magnetic resonance imaging (MRI), and hysteroscopy^3–8^. A niche is present in 60% of women at 2–12 weeks after CS^3,9^. According to our previous study, the prevalence of CSD has reached 43.4% at 6 weeks postpartum^10^. Recently, we became aware of the association between various symptoms and the presence of a niche. Niche-related symptoms and problems include postmenstrual spotting, dysmenorrhea, dyspareunia, chronic pelvic pain, cesarean scar pregnancy, possible infertility, and uterine rupture or placental adhesion during a subsequent pregnancy^3,9,11–18^.

Of all niche-related clinical symptoms, postmenstrual spotting might be the most typical and prevalent symptom. Previous studies have reported that the presence of a niche increases the risk of postmenstrual spotting for more than 2 days^3,9^. Postmenstrual spotting may be derived from the retention of menstrual blood in the niche caused by a mechanical outflow problem^12,13,15,16^ or from the accumulation of blood resulting from impaired uterine contractions at the site of the niche^13^. Additionally, newly formed fragile vessels in the niche may attribute to blood formation in the niche, which is supported by the presence of free blood cells in the endometrial stroma^19^ and hysteroscopic evaluations where small vessels are observed in a majority of patients^20–23^.

Several surgical interventions to treat niche-related symptoms have been developed in recent years, including laparoscopic surgery, hysteroscopic surgery, laparoscopy-assisted hysteroscopic repair, and vaginal repair^21^. A hysteroscopic niche resection can be considered in women with a relatively small niche, with a residual myometrium (RM) of at least 2 mm. If the RM is thinner, a hysteroscopic niche resection is not suitable because of its risk for perforation or bladder injury. The purpose of hysteroscopic surgery is to remove the distal rim to facilitate the outflow of menstrual blood and coagulate the vessels in the niche cavity to reduce blood loss from these fragile vessels. In 2018, Vervoort et.al conducted a randomized controlled study and demonstrated that hysteroscopic niche resection shortened postmenstrual spotting duration by 4 days compared to baseline data and by 3 days compared to the expectant management group^24^. However, the relationship between clinical symptoms, various niche features, and success of hysteroscopic niche resection in terms of reducing spotting has not been elucidated yet. Niche features including morphological characteristics and measurements can be assessed using preoperative imaging techniques such as MRI or TVS^4–6,8^.

To predict the clinical effectiveness of hysteroscopic niche resection, this study aimed to develop and validate a predictive model that incorporated both clinical symptoms and niche morphological characteristics and MRI measurements in patients with postmenstrual spotting and a relatively small niche with a RM of at least 2 mm. Knowledge concerning predictive factors associated with the surgical effects might help in selecting and counseling patients with niche-related spotting and patients for whom hysteroscopic niche resection is considered. To the best of our knowledge, this is the largest study evaluating the prognostic factors for the effectiveness of hysteroscopic niche resection.

## Method and materials

### Study population

This study was approved by the Institutional Review Board of the International Peace Maternity and Child Health Hospital, Shanghai, China: ref. no. (GKLW) 2017–126 obtained on 24/08/2018. The study was registered on Chinese Clinical Trial Register (ChiCTR2000032751) on 09/05/2020. Written informed consent was obtained from all participating women. Participants were informed that they had the right to refuse to participate in the study or withdraw from the study at any time. We confirm that this study was performed in accordance with relevant guidelines and regulations. This prospective cohort study, from July 2017 to September 2019, enrolled all consecutive patients with at least one CS, postmenstrual spotting, and a niche that met the inclusion criteria. The patients were included after they provided informed consent. A niche was diagnosed by MRI and was defined as a pouch in the anterior uterine wall at the site of the cesarean scar of at least 2 mm depth. Postmenstrual spotting was defined as intermenstrual spotting duration ≥ 2 days or brownish discharge for ≥ 2 days immediately after the menstrual period if the duration of menstrual bleeding exceeded 7 days; if the menstrual period was < 7 days, the brownish discharge was considered normal^24^. The following exclusion criteria were applied: (1) RM < 2 mm on MRI, (2) postmenstrual spotting duration < 10 days, (3) pregnancy, (4) malignancy, (5) use of intrauterine devices, (6) uterine disease that could cause abnormal uterine bleeding, such as submucosal fibroids, endometrial polyps, or hyperplasia, (7) use of continuous oral contraceptives or gonadotropin-releasing hormone agonists, and (8) history of coagulation disorders, (9) a history of menstrual irregularities or endocrine disorders before CS.

### Surgical procedure

All patients underwent hysteroscopic niche resection under general anesthesia by skilled gynecologists. These gynecologists were additionally trained in performing hysteroscopic niche resection by one of the experienced gynecologists as described by Vervoort et al.^24^. The operation was conducted using a TCR resectoscope (Ch. 26 model WA22061 with a 12° optic 22001A) equipped with a 3-mm deep and 5-mm wide loop (Olympus Inc., Germany) under 120 mmHg intrauterine pressure using 0.9% NaCl to induce distension of the uterine cavity. The first step was to evaluate and register the features of the niche cavity. Thereafter, a cutting loop was used to resect the distal rim of the niche gradually as described by Vervoort et al.^24^ and coagulate the surface of the cavity of niche superficially by the loop. The resected tissue was sent for histological examination to assess the presence of endometrium in the niche. Additionally, polyps and Nabothian cysts in the niche cavity were resected, if present. The procedure was discontinued if any complications occurred, such as perforations, or if the estimated fluid deficit was > 1 L. In case of an uneventful procedure, patients were discharged on the next day.

### Follow-up and outcome measures

In this study, surgical efficacy was defined as a shortening in postmenstrual spotting duration of at least 3 days at 3-month follow-up compared with baseline. A threshold of 3 days reduction in postmenstrual spotting duration was based on the results of a survey among 50 patients undergoing hysteroscopic niche resection (characteristics were shown in Table S1). In this survey, we evaluated patients’ expected satisfaction, asked them what parameters they considered most important, and requested them to report the minimal days of shortening in postmenstrual spotting duration to be satisfied. In total, 74% of the patients reported that they would be satisfied with a minimal shortening in postmenstrual spotting duration of 3 days. According to this, the patients were divided into the “effective group” (shortening of postmenstrual spotting duration ≥ 3 days) and “ineffective group” (shortening of postmenstrual spotting duration < 3 days).

### Data collection

We recorded patients’ demographic characteristics and reproductive history, and patients were asked to complete questionnaires that contained questions about menstrual characteristics, menstruation-related pain, discomfort of both spotting and menstrual pain on visual analog scale of 0–10, and patient satisfaction with menstruation (5-point Likert scale) at baseline and at 3-month follow-up. In addition, all participants underwent MRI before surgery. The radiologist selected the sagittal plane where the niche subjectively was the largest. Various niche data were measured and recorded (Appendix S1). The size of the niche was measured by its depth, length, thickness of the residual myometrium (TRM), thickness of the adjacent myometrium (TAM), and TRM/TAM ratio (Fig. S1-a1)^25^. Severe defect was defined as TRM/TAM ratio of < 50%)^18,25^. Niche size was also expressed as the maximal area of the niche in the sagittal plane. It was calculated automatically by the software (Siemens Syngo MR Software Package) after manually outlining the niche (Fig. S2). Shape was defined as linear, triangle, or irregular (Fig. S3)^8^. To laterally describe the shape of the niche, various angles, including angles α, β, and γ (Fig. S4), and the projection point of the apex of the niche on the line between the endpoint of the upper edge and the endpoint of the lower edge of the niche (Fig. S5) were measured. In addition, presence of a lateral branch, polyp, or a cyst in relation with the niche was also registered (Fig. S6). Apart from its size and shape, the location of the niche in relation to the internal os (distance between the apex of the niche and the most distal point of the niche at the base of the niche) was measured (Fig. S1-a2). The position of the niche in relation to the position of the uterus (Fig. S1-a2) and the position of the corpus in relation to the cervix (Fig. S7) were expressed with angle θ. As another important parameter of the niche, width was measured in the coronal plane (Fig. S8). Moreover, postoperative data including duration of the operation, fluid loss, complications, and presence of endometrium (identified by pathology) in the niche, were recorded.

### Statistical analysis

All patients who completed the questionnaire at baseline and at 3-month follow-up were included in the analyses and for the development of the predictive model. All statistical analyses were performed using the R Statistical Computing software (version 3.5.1, https://www.Rproject.org). One-sample Kolmogorov–Smirnov test was used for normality test. Follow-up data were compared with baseline data using paired t-tests if normally distributed and equally varied; otherwise, paired non-parametric tests were used. A *p* value of < 0.05 was considered statistically significant.

### Development of the model including preoperative data only (Model 1)

Preoperative clinical variables and MRI characteristics of the niche were used to develop a predictive model, including only preoperative data (Model 1), of the clinical effectiveness of hysteroscopic niche resection. First, descriptive statistics analysis was performed using frequencies and percentages for categorical variables and means and standard deviation (SD) or median (interquartile range, IQR) for continuous variables. The multicollinearity among all variables was evaluated by variance inflation factor (VIF); VIF < 10 was considered acceptable. Univariate relationships of the potential predictive factors between the effective and ineffective groups were evaluated by chi-squared or exact Fisher’s test for categorical variables and Student’s *t* test or Mann–Whitney *U* test according to the data distribution for continuous variables. Following Lemeshow’s statistical criteria, variables with *p* value < 0.25were included in the multivariate binary logistic regression model using stepwise elimination. A nomogram was used as a presentation of the predictive model on the basis of the multivariable logistic analysis.

### Performance of the model

We evaluated the performance of the predictive model with respect to three main features: discrimination, calibration, and clinical utility. The discrimination ability was evaluated by the area under the receiver operating characteristic (ROC) curve (AUC). In addition, model calibration was assessed with calibration plot accompanied with the Hosmer–Lemeshow test^26^. Furthermore, to estimate the clinical utility of this model, decision curve analysis (DCA) was performed by calculating the net benefits for a range of threshold probabilities in the validation dataset. Additionally, inner validation was performed using tenfold cross-validation.

### Development of a model adding histological findings (Model 2)

The presence of endometrium in the defect was assessed by histological examination of the resected tissue. To assess the association between the presence of endometrium in the defect and the effect of the hysteroscopic niche resection, the incremental value of the histologic finding as an additional candidate predictor was evaluated (Model 2), and the AUC and calibration curve were derived. The decision curve was also plotted for the model after the addition of histologic findings.

### Ethics approval

This study was approved by the Institutional Review Board of the International Peace Maternity and Child Health Hospital, Shanghai, China: ref. no. (GKLW) 2017-126 obtained on 24/08/2018. The study was registered on Chinese Clinical Trial Register (ChiCTR2000032751) on 09/05/2020. Written informed consent was obtained from all participating women. Participants were informed that they had the right to refuse to participate in the study or withdraw from the study at any time.

## Results

**Figure 1:**
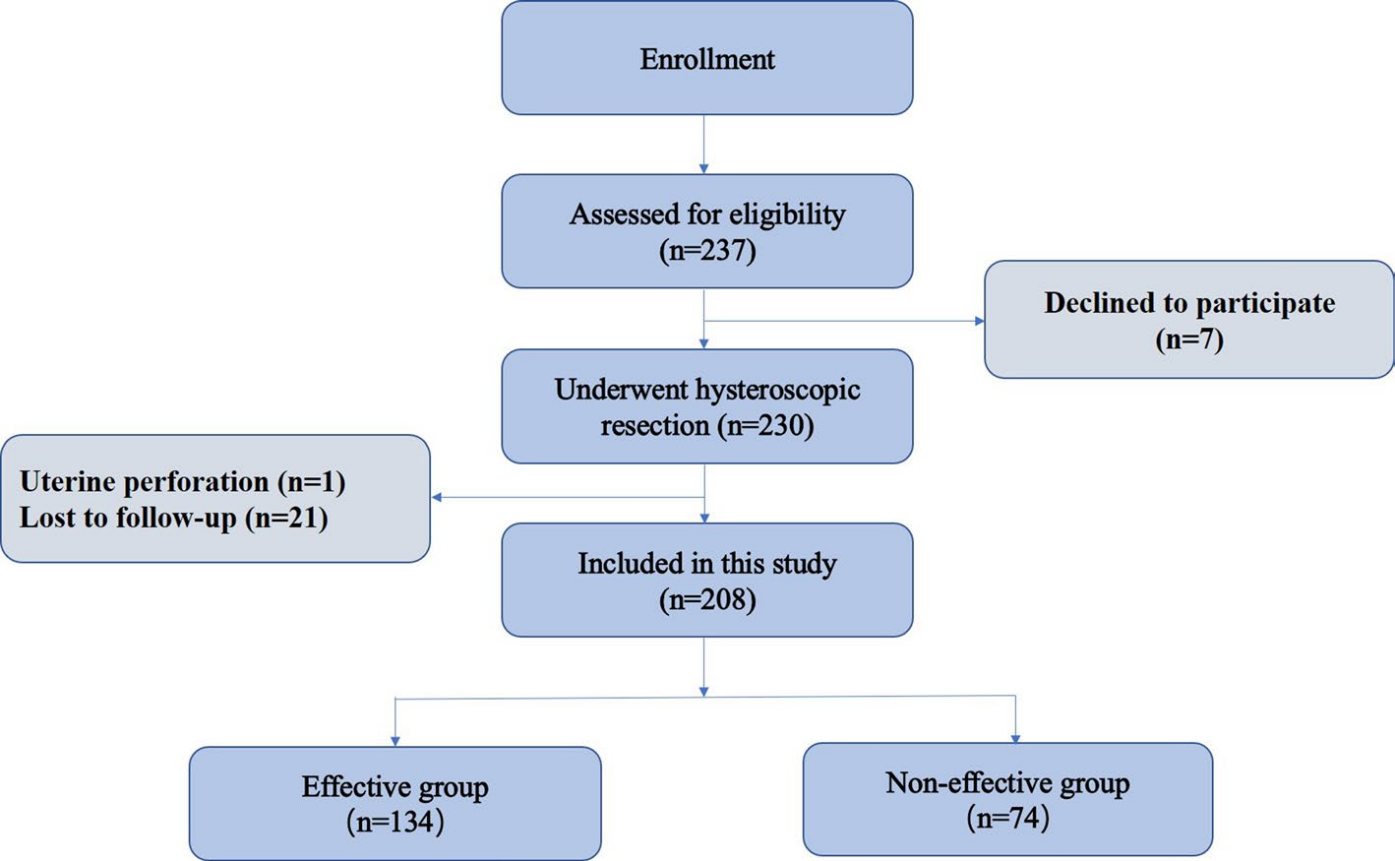
Flow chart.

The flow chart of population selection is shown in Fig. 1. Of the 230 women who underwent hysteroscopic niche resection, 208 women completed both baseline and follow-up examination. Baseline data and results at the 3-month follow-up are shown in Table 1. The median duration of menstruation shortened from 13 days (IQR 10–15 days) to 8 days (IQR 7–11 days) at 3 months after surgery (*p* < 0.01). The median duration of postmenstrual spotting improved from 6 days (IQR 3–8 days) to 1 day (IQR 0–4 days) at the 3-month follow-up (*p* < 0.01). The VAS score of discomfort due to the spotting decreased from 6 (IQR 3–8) to 2 (IQR 1–4) statistically significantly at the 3-month follow-up (*p* < 0.01). Furthermore, women’s satisfaction with her menstruation improved after hysteroscopic niche resection from 2.10 ± 1.05 to 3.53 ± 1.41 (*p* < 0.01). Apart from one case of uterine perforation, no other complications occurred, and all other cases were completely resected without premature termination of the procedure. At 3 months after surgery, 134 patients had ≥ 3 days reduction of postmenstrual spotting duration (effective group), 74 patients < 3 days reduction of spotting duration (ineffective group). Table 2 compares the baseline characteristics, preoperative bleeding characteristics, and histologic results between the effective and ineffective groups. MRI characteristics of the two groups were also compared (Table 3). Multicollinearity detection indicates no multicollinearity between all independent variables (all VIF < 10).

**Table 1:** Characteristics of patients with niche before and 3 months after hysteroscopic surgery.

Variables	Before surgery (n = 208)	After surgery (n = 208)	p value
**Bleeding characteristics**			
Duration of menstruation (days)	13 (10,15)	8 (7, 11)	< 0.01
Postmenstrual spotting (days)	6 (3, 8)	1 (0, 4)	< 0.01
Spotting at the end of the menstruation (days)	6 (3, 8)	1 (0, 4)	< 0.01
Intermenstrual spotting (days)	0 (0, 0)	0 (0, 0)	0.82
**Discomfort score from spotting (0–10)**	6 (5, 7)	2 (1, 4)	< 0.01
**Dysmenorrhea score (0–10)**	0 (0, 3)	0 (0, 3)	1.00
**Chronic pelvic pain score (0–10)**	1 (0, 3)	0 (0, 3)	0.85
**Women's satisfaction with her menstruation (Five-point likert scale) (1–5)**	2.10 ± 1.05	3.53 ± 1.41	< 0.01

Data are reported as mean ± standard deviation or as median (interquartile range, IQR) for each parameter.

Postmenstrual spotting days = the sum of the number of days spotting at the end of the menstruation and the number of days of intermenstrual spotting.

**Table 2:** Baseline characteristics and histologic examination in the two groups.

Variables	Effective group (n = 134)	Non-effective group (n = 74)	p value
**Age (years)**	34.52 ± 4.53	34.70 ± 4.54	0.78
**Gravidity**	2 (1,3)	2 (1,3)	0.63
**Parity**	1.45 ± 0.62	1.34 ± 0.58	0.21
**Number of caesarean sections**	1 (1,2)	1 (1,2)	0.68
**Preoperative menstrual duration (days)**	14 (12,15)	12 (10,14)	< 0.01
**Preoperative dysmenorrhea score (0–10)**	0 (0,3)	0 (0,2)	0.13
**Preoperative chronic pelvic pain score (0–10)**	0 (0,2)	0 (0,2)	0.22
**Preoperative discomfort score from spotting (0–10)**	6 (5,8)	5 (4,7)	0.02
**Preoperative five-point likert scale (1–5)**	2.06 ± 1.02	2.39 ± 1.02	0.02
**Histologic examination presence of endometrium in the niche**			0.08
YES	72 (53.73%)	30 (40.54%)	
NO	62 (46.27%)	44 (59.46%)	

Data are reported as mean ± standard deviation or median (interquartile range, IQR) or as n (valid percentage).

Preoperative characteristics defined as after CS prior to hysteroscopic surgery.

**Table 3:** Preoperative MRI findings in the two groups.

Variables	Effective group (n = 134)	Non-effective group (n = 74)	p value
**Length (mm)**			0.05
< 9	70 (52.24%)	28 (37.84%)	
≥ 9	64 (47.76%)	46 (62.16%)	
**Depth (mm)**			0.02
< 5	61 (45.52%)	21 (28.38%)	
≥ 5	74 (55.22%)	53 (71.62%)	
**Width (mm)**			0.47
< 12	71 (52.99%)	43 (58.11%)	
≥ 12	63 (47.01%)	31 (41.89%)	
**TRM (mm)**			< 0.01
< 2.2	12 (8.96%)	27 (36.49%)	
≥ 2.2	122 (91.04%)	47 (63.51%)	
**TAM (mm)**			0.27
< 9	52 (38.81%)	23 (31.08%)	
≥ 9	82 (61.19%)	51 (68.92%)	
**The degree of severity of the defect (TRM/TAM)**			< 0.01
Mild (≥ 50%)	37 (27.61%)	4 (5.41%)	
Severe (< 50%)	97 (72.39%)	70 (94.59%)	
**Angle (α)**			0.47
< 70°	90 (67.16%)	46 (62.16%)	
≥ 70°	44 (32.84%)	28 (37.84%)	
**Angle (β)**			0.78
< 70°	57 (42.54%)	30 (40.54%)	
≥ 70°	77 (57.46%)	44 (59.46%)	
**Angle (γ)**			< 0.01
< 90°	66 (49.25%)	19 (25.68%)	
≥ 90°	68 (50.75%)	55 (74.32%)	
**Angle (θ)**			0.67
≤ 90°	9 (6.72%)	2 (2.70%)	
91°–180°	52 (38.81%)	31 (41.89%)	
181°–270°	58 (43.28%)	32 (43.24%)	
≥ 271°	15 (11.19%)	9 (12.16%)	
**Area (mm**^**2**^**)**			< 0.01
< 50	126 (94.03%)	51 (68.92%)	
≥ 50	8 (5.97%)	23 (31.08%)	
**The projection point of the C on the line between A and B**			0.33
Within A and B	109 (81.34%)	56 (75.68%)	
Outside A and B	25 (18.66%)	18 (24.32%)	
**The distance between the lower point of niche to the internal cervical os (mm)**			0.46
< 3	85 (63.43%)	43 (58.11%)	
≥ 3	49 (36.57%)	31 (41.89%)	
**The distance between the lowest demarcation of niche to the internal cervical os (mm)**			0.55
< 5	51 (38.06%)	25 (33.78%)	
≥ 5	83 (61.94%)	49 (66.22%)	
**Shape**			0.09
Linear	13 (9.70%)	7 (9.46%)	
Triangle	33 (24.63%)	9 (12.16%)	
Irregular rectangular	88 (65.67%)	58 (78.38%)	
**Lateral branch**			0.18
YES	17 (12.68%)	5 (6.76%)	
NO	117 (87.31%)	69 (93.24%)	
**Polyp in the cavity of niche**			0.57
YES	8 (5.98%)	6 (8.11%)	
NO	128 (95.52%)	68 (91.89%)	
**Cyst in the cavity of niche**			0.98
YES	11 (8.21%)	6 (8.11%)	
NO	123 (91.79%)	68 (91.89%)	

CS = cesarean section; MRI = magnetic resonance image; TRM = thickness of the residual myometrium; TAM = thickness of the adjacent myometrium; TRM/TAM = the ratio of myometrial thickness at the scar to the thickness of adjacent myometrium.

Angle α was defined as the angle of the upper margin of the defect; Angle β was defined as the angle of lower margin of the defect; Angle γ was defined as the angle of the apex of the defect; Angle θ was defined as angle between the cervical axis and the axis of uterine corpus.

A: the endpoint of upper edge of niche on MRI; B: the endpoint of lower edge of niche on MRI; C: the apex of niche on MRI.

Data are reported as mean ± standard deviation as median (interquartile range, IQR) or as n (valid percentage).

### Results of the predictive model including preoperative factors only (Model 1)

**Figure 2:**
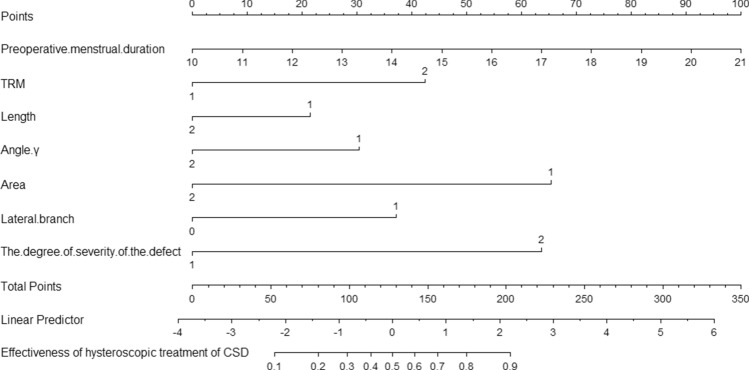
Nomogram of the predictive model. TRM (1: ≥ 2.2 mm; 2: < 2.2 mm); Length (1: < 9 mm; 2: ≥ 9 mm); Angle γ (1: < 90°; 2: ≥ 90°); Area (1: < 50mm^2^;2: ≥ 50 mm^2^); Lateral branch (0: No; 1:Yes); The degree of severity of the niche (1: Severe (TRM/TAM < 50%) ; 2: Mild (TRM/TAM ≥ 50%)).

Table 4 displays the final multivariable logistic regression model (Model 1). In total, seven independent predictors of the effectiveness of hysteroscopic niche resection were found: preoperative menstrual duration, angle γ, TRM, length, area, lateral branch, and severity of the defect. The model that incorporated the above independent predictive factors was developed and is presented as a nomogram (Fig. 2).

**Table 4:** Risk Factors for the effectiveness of hysteroscopic surgery for niche.

	Model 1			Model 2		
Intercept and Variable	β	Odds Ratio (95% CI)	p value	β	Odds Ratio (95% CI)	p value
Intercept	− 0.77	0.46 (0.02–9.94)	0.62	− 2.19	0.11 (0.03–3.16)	0.19
**Preoperative menstrual duration**	0.27	1.30 (1.22–1.52)	< 0.01	0.28	1.32 (1.14–1.55)	< 0.01
**TRM (mm)**	1.24	3.45 (1.43–8.33)	< 0.01	1.36	3.91 (1.58–9.67)	< 0.01
**Length (mm)**	− 0.63	0.53 (0.26–1.08)	0.08	− 0.58	0.58 (0.27–1.14)	0.11
**Angle (γ)**	− 0.89	0.41 (0.20–0.86)	0.02	− 1.12	0.32 (0.15–0.71)	< 0.01
**Area (mm**^**2**^**)**	− 1.91	0.15 (0.05–0.43)	< 0.01	− 1.90	0.15 (0.05–0.44)	< 0.01
**Lateral branch**	1.09	2.97 (0.82–10.67)	0.03	0.1	2.99 (0.80–11.17)	0.10
**The degree of severity of the niche**	1.86	6.41 (1.85–22.24)	< 0.01	1.4	6.15 (1.70–22.24)	0.01
**Presence of endometrium in the niche**	NA	NA		0.85	2.35 (1.10–4.99)	0.03
**AUC**		0.834			0.826	

TRM = thickness of the residual myometrium; AUC = the area under the receiver operating characteristic curve.

### Performance of Model 1

**Figure 3:**
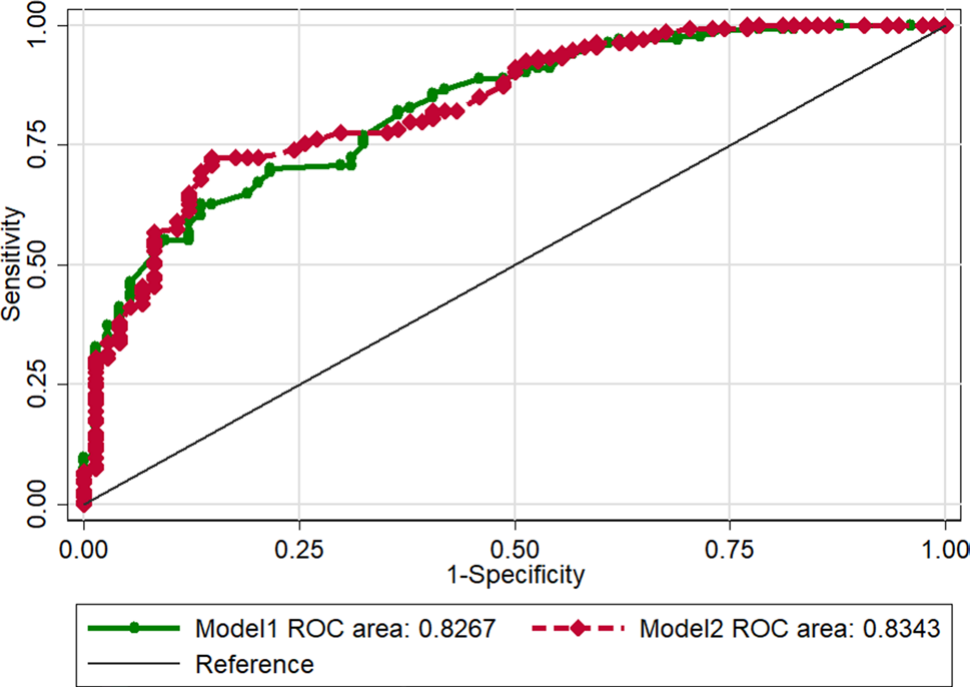
Receiver operating characteristic (ROC) curves of the predictive model. Area under the ROC curve to determine the predictive ability of the model, representing the sensitivity on the ordinate axis and specificity in the abscissa. The green line represented the model 1. The red line represented the model 2.

**Figure 4:**
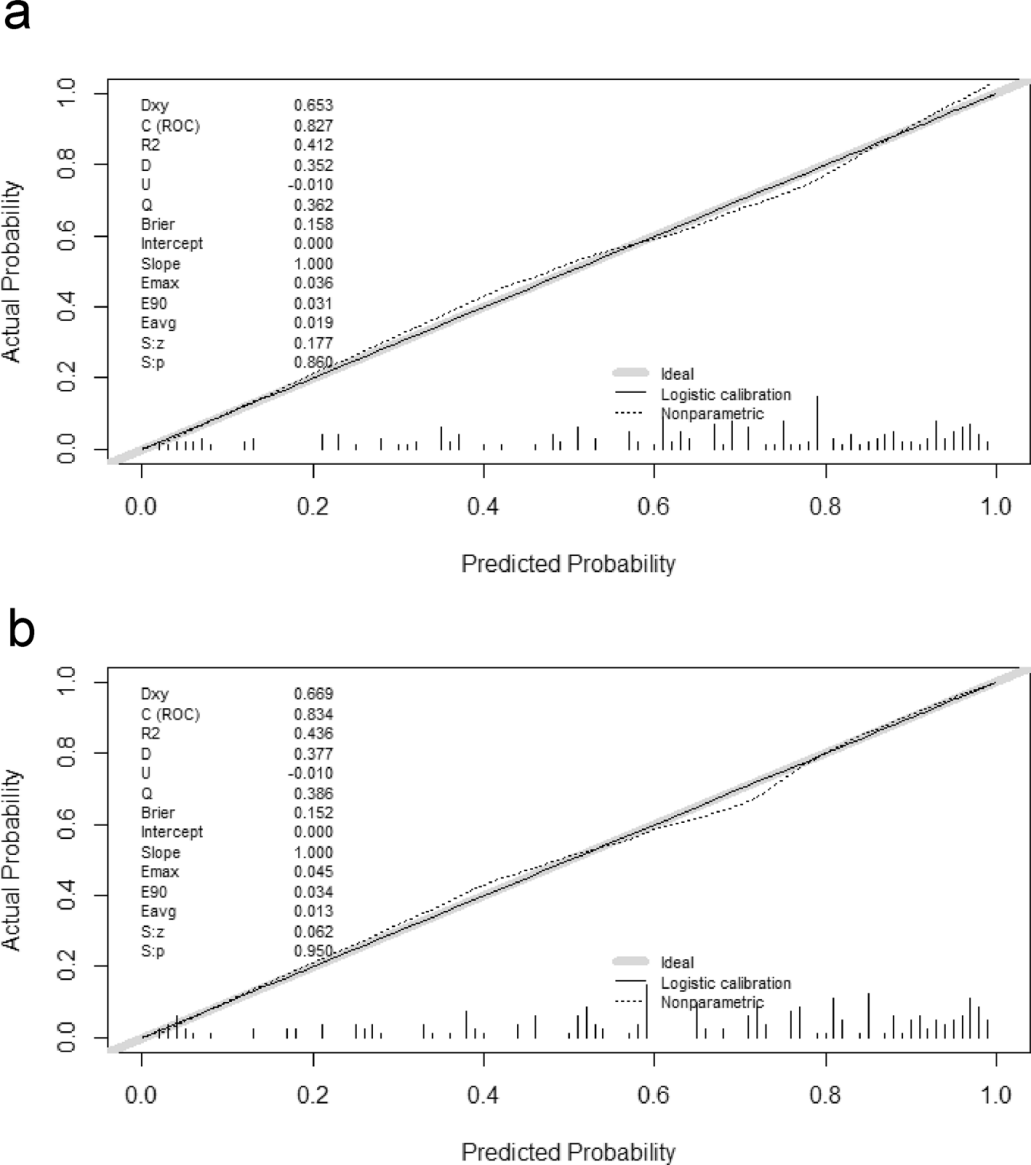
Calibration plot of the predictive model. Calibration plot depicted the agreement between the predicted effective rate of hysteroscopic surgery and observed outcomes of effective rate. The y-axis represented the actual effective rate. The x-axis represented the predicted effective rate. The diagonal solid line represents a perfect prediction by an ideal model. The dotted line represented the performance of the model, of which a closer fit to the diagonal solid line represented a better prediction. (**a**) represented the model 1. (**b**) represented the model 2.

**Figure 5:**
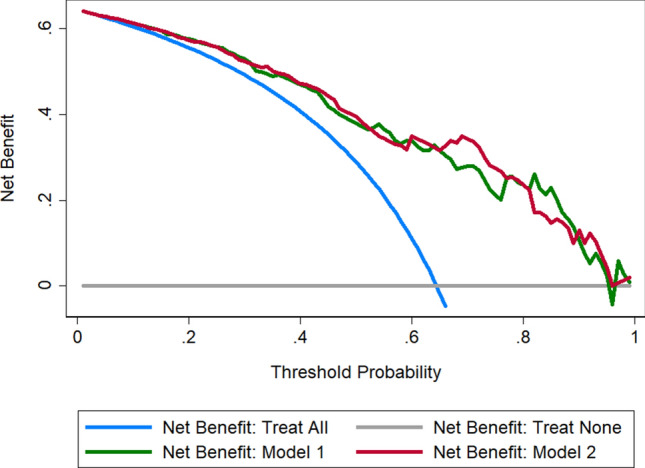
Decision curve analysis (DCA) for the predictive model. The y-axis measures the net benefit. The blue line represented the assumption that all patients can benefit from hysteroscopic surgery. The gray line represented the assumption that no patients can benefit from hysteroscopic surgery. The green line represented the model 1. The red line represented the model 2. The net benefit was calculated by subtracting the proportion of all patients who are false positive from the proportion who are true positive, weighting by the relative harm of forgoing treatment compared with the negative consequences of an unnecessary treatment. Threshold probability is where the expected benefit of treatment is equal to the expected benefit of avoiding treatment.

Discrimination ROC curves are plotted in Fig. 3. The performance of the model was good with an AUC-ROC of 0.83. The calibration plot is presented in Fig. 4A. The calibration plot shows adequate calibration, with no significant difference between the predicted and observed probability (*p* = 0.86). The DCA for the model is presented in Fig. 5, as nomogram to predict the effectiveness of hysteroscopic niche resection which benefits more than the treat-all-patients scheme or the treat-none scheme, suggesting that the nomogram is clinically useful. Furthermore, the tenfold cross-validation shows that the error rate of this predictive model (Model 1) is 24.90%.

### Results of predictive model after adding the histological result (Model 2)

The predictive model after the addition of histologic findings is shown in Table 4 (Model 2). Although a slightly higher ROC-AUC was observed for the model containing histologic findings, integration of the histologic findings into the predictive model did not improve the predictive performance significantly (Fig. 3, AUC: 0.827 vs 0.834, *p* = 0.47). The calibration curve of Model 2 for the effectiveness of hysteroscopic niche resection demonstrates good agreement between prediction and observation (Fig. 4B). According to the DCA, net benefit was comparable on the basis of Model 1 and Model 2 (with histologic result integrated, Fig. 5).

## Discussion

### Main findings

We developed and validated a model to predict the effectiveness of hysteroscopic niche resection in shortening the postmenstrual spotting duration (> 3 days) by integrating preoperative variables, including MRI characteristics, and the presence of endometrium during histological examination. Key factors that were integrated in this predictive model were menstrual duration at baseline and some MRI characteristics evaluated in the sagittal plane (TRM, length, presence of a branch, niche area, TRM/TAM ratio and angle γ). The addition of the histological findings for evaluating the presence of the endometrium was not of beneficial value. The performance of the model in predicting the reduction of postmenstrual spotting (≥ 3 days or < 3 days) was good. The effectiveness of the hysteroscopic niche resection improved if the nomogram was used, underlining its clinical relevance.

### Strengths and limitations

#### Strengths

Patients were included consecutively, and the response rate was high, reducing the risk of selection bias. In addition, in this study, hysteroscopic niche resections were executed by skilled surgeons in a standardized manner. Finally, despite the limited sample size, internal validation was performed using tenfold cross-validation. The advantage of this method over repeated random sub-sampling is that all observations were used for both training and validation, and each observation was used for validation once.

#### Limitations

There are some limitations of this study. First, we were unable to perform external validation of the models because of the small sample size. The accuracy of the model might be affected by various clinical situations. Therefore, we need to be cautious in generalizing the implications of the model before it is utilized in different hospitals and countries. What’s more, when doing external validation in different hospitals or country, the hysteroscopic technique by which the niche was treated can be added since 16 Fr mini-resectoscope has been used recently. Second, the variables included in our study may not be comprehensive enough, such as whether there are valve-like low rim of the niche which may be a potential factor affecting the efficacy of hysteroscopic resection and need to be improved in future studies. What’s more, to make the current model as simple as possible, we only included two-dimensional measurements of MRI planes that were taken with a 4-mm interval; this may not fully reflect the three-dimensional morphology of the niche. The additional value of a smaller interval and measurements taken in all planes need to be studied. Similarly, real-time 2D and 3D ultrasound evaluation with or without saline instillation needs to be employed as these are mostly used in other countries in the evaluation of niches^2,11^.

#### Interpretation

The outcomes necessary to call a hysteroscopic niche resection effective are still debatable. Thus, in this study, we surveyed women who planned to undergo hysteroscopic niche resection to determine their expectations, and we consequently found that a minimal reduction of 3 days in the duration of postmenstrual spotting was reported by the majority of the patients to be satisfied with the outcomes of hysteroscopic niche resection. This outcome is also in line with the primary outcome and results of a previous randomized trial evaluating the effectiveness of hysteroscopic niche resection. In this study, the median reduction in the duration of postmenstrual spotting after a hysteroscopic niche resection in comparison to a control group was 3 days^24^. However, given the small sample size, Vervoort et al. were unable to determine prognostic factors for a successful niche resection. Due to the small sample size of our preliminary investigation which cannot represent the expectations of all patients, we must admit the limitations of our study and more attempts on other indicators and cut-off values can be made in the future studies.

Our study suggested that a longer preoperative menstrual duration was associated with a higher change on the reduction of postmenstrual spotting duration of at least 3 days. This phenomenon might be attributed to the fact that the more obvious are the symptoms of menstrual disorders, the more typical is the cause of bleeding, and the easier the operation will solve this problem.

As we discussed previously, we used MRI for the preoperative evaluation of the niche, whereas most European studies used TVS with or without saline instillation. Recently, the European niche taskforce group published a guideline for standardized measurement and reporting of niches^2^. This guideline advocated reporting niche length, depth, width, TRM, TAM, and the presence of branches. Different definitions are used to describe a large niche; for example, Osser et al*.* defined a large defect as scar myometrial thickness of < 2.2 mm on TVS or < 2.5 mm on sonohysterogram^27^, while Ofli-Yebovi et al*.* defined severe defect as TRM/TAM ratio of < 50%^25^.

As regards MRI, there are no clear guidelines in the evaluation and measurement of niches. In the current study, we measured various parameters using MRI; apart from the factors advised by the European niche taskforce group, we measured the area of the niche, various angles between the niche and the uterus, and the niche location. Based on our model, various parameters were of prognostic value in the prediction of a reduction in postmenstrual spotting duration of at least 3 days following hysteroscopic niche resection, and these include smaller TRM, longer length, bigger area, larger angle γ, and presence of a severe defect, which were all associated with poorer effectiveness of the hysteroscopic niche resection. Thus, based on our data, hysteroscopic niche resection was effective in small niches but not for case of a relatively large niche. In other words, we should not perform hysteroscopic niche resection on patients with large niches because it is ineffective. For larger niches, other treatment methods may be considered. In addition, since this study was not a comparative study, it could only answer the question that hysteroscopic niche resection was suitable for small niches. More comparative studies are needed to answer the question of which surgical approach is more effective for a relatively small niche.

In general, performing complete resection with full exposure of the entire niche is difficult, and the increased risk of bladder perforation or injury in cases of thin RM may induce incomplete resection. The presence of a lateral branch was found to improve the result in a higher change on the reduction of postmenstrual spotting duration of at least 3 days. We expected that a branch would reduce the overview of the niche, but apparently, this is not the case. On the contrary, accumulation of blood in a branch may play a role in blood retention following a normal menstruation, leading to spotting and widening of the opening, and resection of this branch may facilitate menstrual outflow and consequently reduce postmenstrual spotting.

The presence of endometrium in the defect can be determined through histologic examination after surgery. In 1995, Morris analyzed 51 uterine samples of women who underwent hysterectomy for abnormal uterine bleeding and with history of CS and found that 65% of the samples present with free red blood cells in the endometrial stroma of the scar suggesting recent hemorrhage^19^. Based on this, we speculate that the accumulated blood may be produced in situ for patients with niche. Therefore, we expected that the presence of endometrium in the defect was associated with improved reduction in postmenstrual spotting duration following a hysteroscopic niche resection. However, the addition of histologic result to the prediction model did not improve the performance of the model.

Based on our data, we advise using a model that includes only data that can be preoperatively obtained, such as menstrual duration and MRI findings. By this, we developed a nomogram that can be used to predict the effectiveness of a hysteroscopic niche resection, which enables us to identify patients who may benefit from the treatment. Future studies are needed to evaluate the external validity of this model and to evaluate whether TVS measurements could be used instead of MRI measurements.

## Conclusions

Our predictive model might be clinically useful because it can be used in counseling patients and enables clinicians to select patients eligible for hysteroscopic niche resection.

## Supplementary information

Supplementary Information.

## References

[1] Naji O (2012). Standardized approach for imaging and measuring Cesarean section scars using ultrasonography. Ultrasound Obstet. Gynecol..

[2] Jordans IPM (2019). Sonographic examination of uterine niche in non-pregnant women: A modified Delphi procedure. Ultrasound Obstet. Gynecol..

[3] van der Voet LF, Bij de Vaate AM, Veersema S, Brolmann HA, Huirne JA (2014). Long-term complications of caesarean section The niche in the scar: A prospective cohort study on niche prevalence and its relation to abnormal uterine bleeding. BJOG.

[4] Glavind J, Madsen LD, Uldbjerg N, Dueholm M (2016). Cesarean section scar measurements in non-pregnant women using three-dimensional ultrasound: A repeatability study. Eur. J. Obstet. Gynecol. Reprod. Biol..

[5] Baranov A, Gunnarsson G, Salvesen KA, Isberg PE, Vikhareva O (2016). Assessment of Cesarean hysterotomy scar in non-pregnant women: Reliability of transvaginal sonography with and without contrast enhancement. Ultrasound Obstet. Gynecol..

[6] Fiocchi F (2015). Transvaginal ultrasound assessment of uterine scar after previous caesarean section: Comparison with 3T-magnetic resonance diffusion tensor imaging. Radiol. Med. (Torino).

[7] van der Voet LLF (2017). Niches after cesarean section in a population seeking hysteroscopic sterilization. Eur. J. Obstet. Gynecol. Reprod. Biol..

[8] Wong WSF, Fung WT (2018). Magnetic resonance imaging in the evaluation of cesarean scar defect. Gynecol. Minim. Invasive Ther..

[9] Bij de Vaate AJ (2011). Ultrasound evaluation of the Cesarean scar: Relation between a niche and postmenstrual spotting. Ultrasound Obstet. Gynecol..

[10] Pan H (2019). The prevalence and risk predictors of cesarean scar defect at 6 weeks postpartum in Shanghai, China: A prospective cohort study. Acta Obstet. Gynecol. Scand..

[11] Bij de Vaate AJ (2014). Prevalence, potential risk factors for development and symptoms related to the presence of uterine niches following Cesarean section: Systematic review. Ultrasound Obstet. Gynecol..

[12] Fabres C (2003). The cesarean delivery scar pouch: Clinical implications and diagnostic correlation between transvaginal sonography and hysteroscopy. J. Ultrasound Med..

[13] Thurmond AS, Harvey WJ, Smith SA (1999). Cesarean section scar as a cause of abnormal vaginal bleeding: Diagnosis by sonohysterography. J. Ultrasound. Med..

[14] Wang CB (2009). Cesarean scar defect: Correlation between Cesarean section number, defect size, clinical symptoms and uterine position. Ultrasound Obstet. Gynecol..

[15] Erickson SS, Van Voorhis BJ (1999). Intermenstrual bleeding secondary to cesarean scar diverticuli: Report of three cases. Obstet. Gynecol..

[16] Van Horenbeeck A, Temmerman M, Dhont M (2003). Cesarean scar dehiscence and irregular uterine bleeding. Obstet. Gynecol..

[17] Pomorski M, Fuchs T, Zimmer M (2014). Prediction of uterine dehiscence using ultrasonographic parameters of cesarean section scar in the nonpregnant uterus: A prospective observational study. BMC Pregnancy Childbirth.

[18] Timor-Tritsch IE (2014). Cesarean scar pregnancy is a precursor of morbidly adherent placenta. Ultrasound Obstet. Gynecol..

[19] Morris H (1995). Surgical pathology of the lower uterine segment caesarean section scar: Is the scar a source of clinical symptoms?. Int. J. Gynecol. Pathol..

[20] Fabres C (2005). Surgical treatment and follow-up of women with intermenstrual bleeding due to cesarean section scar defect. J. Minim. Invasive Gynecol..

[21] van der Voet LF (2014). Minimally invasive therapy for gynaecological symptoms related to a niche in the caesarean scar: A systematic review. BJOG.

[22] Gubbini G, Casadio P, Marra E (2008). Resectoscopic correction of the "isthmocele" in women with postmenstrual abnormal uterine bleeding and secondary infertility. J. Minim. Invasive Gynecol..

[23] Vervoort AJ (2015). Why do niches develop in Caesarean uterine scars? Hypotheses on the aetiology of niche development. Hum. Reprod..

[24] Vervoort A (2018). Hysteroscopic resection of a uterine caesarean scar defect (niche) in women with postmenstrual spotting: A randomised controlled trial. BJOG.

[25] Ofili-Yebovi D (2008). Deficient lower-segment Cesarean section scars: Prevalence and risk factors. Ultrasound Obstet. Gynecol..

[26] Kramer AA, Zimmerman JE (2007). Assessing the calibration of mortality benchmarks in critical care: The Hosmer–Lemeshow test revisited. Crit. Care Med..

[27] Osser OV, Jokubkiene L, Valentin L (2009). High prevalence of defects in Cesarean section scars at transvaginal ultrasound examination. Ultrasound Obstet. Gynecol..

