# Supplementary material for: Effectiveness of hysteroscopic resection of a uterine caesarean niche can be predicted: a prospective cohort study
Source: Sci Rep. 2020 Oct 15;10:17424. doi: 10.1038/s41598-020-74622-8 (PMC7566491; doi:10.1038/s41598-020-74622-8)
Supplement: Supplementary file 1 — Supplementary Information. [file 41598_2020_74622_MOESM1_ESM.pdf]

Effectiveness of hysteroscopic resection of a uterine caesarean niche can be predicted: a prospective cohort study

Qian Zhu[1,2,§], Xiaoqing He[1,2,§], Ling Jiang[2,3], Guiling Liang[1,2], Chenfeng Zhu[1,2], Hongjie Pan[4], Jian Zhang[1,2,*] and Judith Anna Huirne[5]

1 Department of Obstetrics and Gynecology, International Peace Maternity and Child Health Hospital, School of Medicine, Shanghai Jiaotong University, Shanghai, China

2 Shanghai Key Laboratory Embryo Original Diseases, Shanghai, China

3 Department of radiology, International Peace Maternity and Child Health Hospital, School of Medicine, Shanghai Jiaotong University, Shanghai, China

4 Department of Obstetrics and Gynecology, Sir Run Run Shaw Hospital, School of Medicine, Zhejiang University, Hangzhou, China

5 Department of Obstetrics and Gynaecology Amsterdam University Medical Centers, Amsterdam Reproduction and Development research institute, Amsterdam, the Netherlands

§ Co-first author

* Corresponding author:

Jian Zhang, Department of Obstetrics and Gynecology, International Peace Maternity and Child Health Hospital, School of Medicine, Shanghai Jiaotong University, Shanghai, China;

Email: zhangjian_ipmch@sjtu.edu.cn

Figure S1. Niche measurement in the sagittal plane

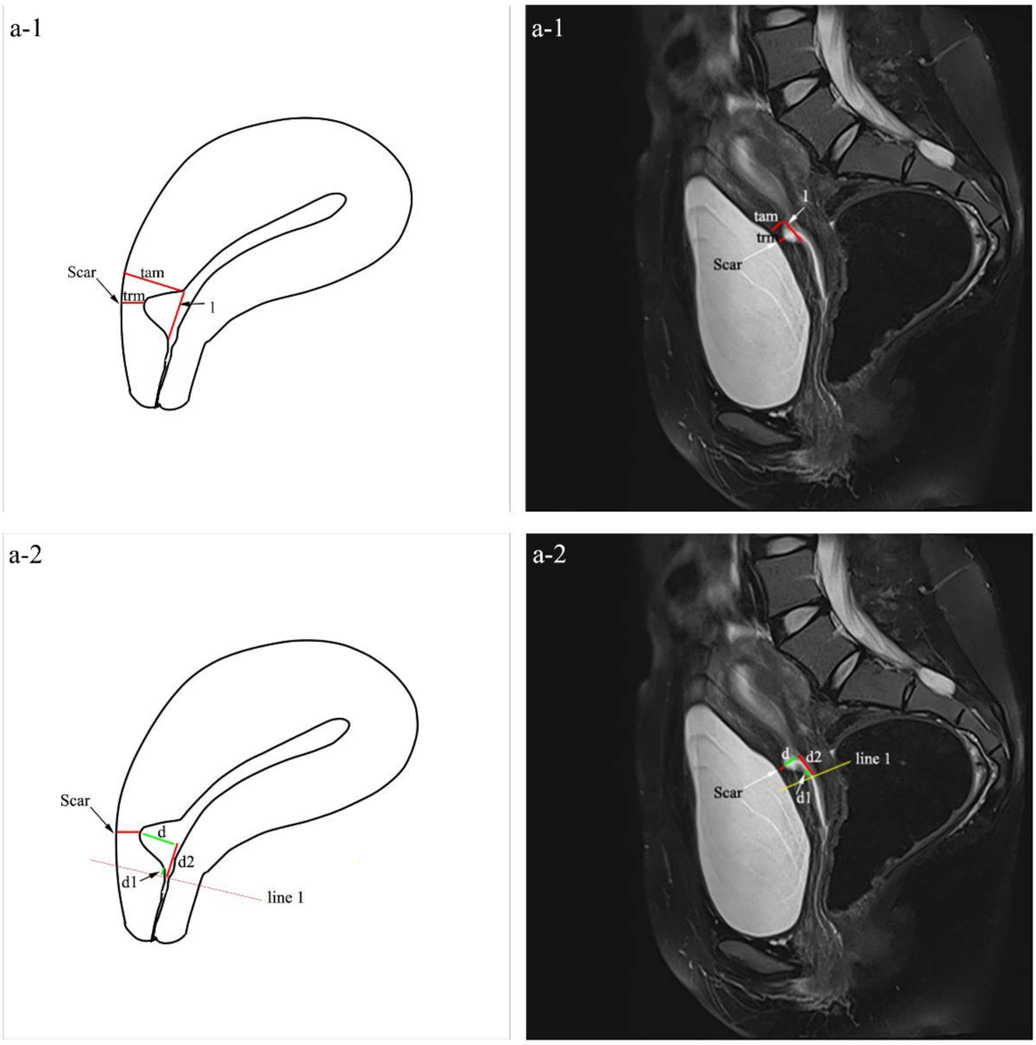

Niche measurement in the sagittal plane: (a) the apex of niche located within the endpoint of upper and lower rim; (l) the length of niche, measured from the upper rim to the lower rim of niche; (d) the depth of niche,measured from the usual limit of uterine limit to the apex of niche; (trm) the thickness of residual myometrium, measured from the apex of niche until the serosa; (tam) the thickness of adjacent myometrium, measured from the upper endpoint of niche to the serosa; (line 1) the level of the internal cervical os, defined as the level where there is a slight narrowing of uterus be tween corpus and the cervix; (line 2) a line through point C and perpendicular to the length of niche; (Scar) the scar of cesarean is defined as the thinest portion of the lower uterine segment; (d1) measured from the lower point of niche to the internal cervical os; (d2) measured from the lowest demarcation to the internal cervical os;

a1: Length (l), TRM, TAM;

a2: Depth (d), Distance between the lower point of niche to the internal cervical os (d1); Distance between the lowest demarcation to the internal cervical os (d2).

Figure S2. The area of niche measurement.

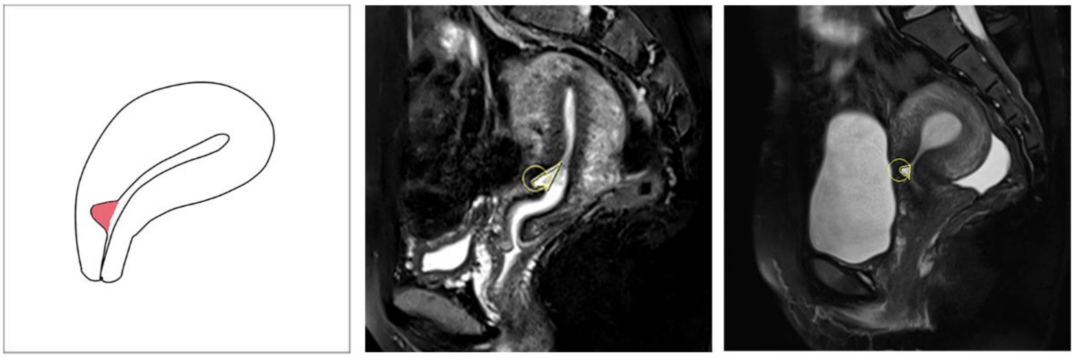

The area of niche measurement in the MRI

The irregular area of niche is defined as the biggest area of the niche in the sagittal palnes.

Figure S3. The shapes of niche.

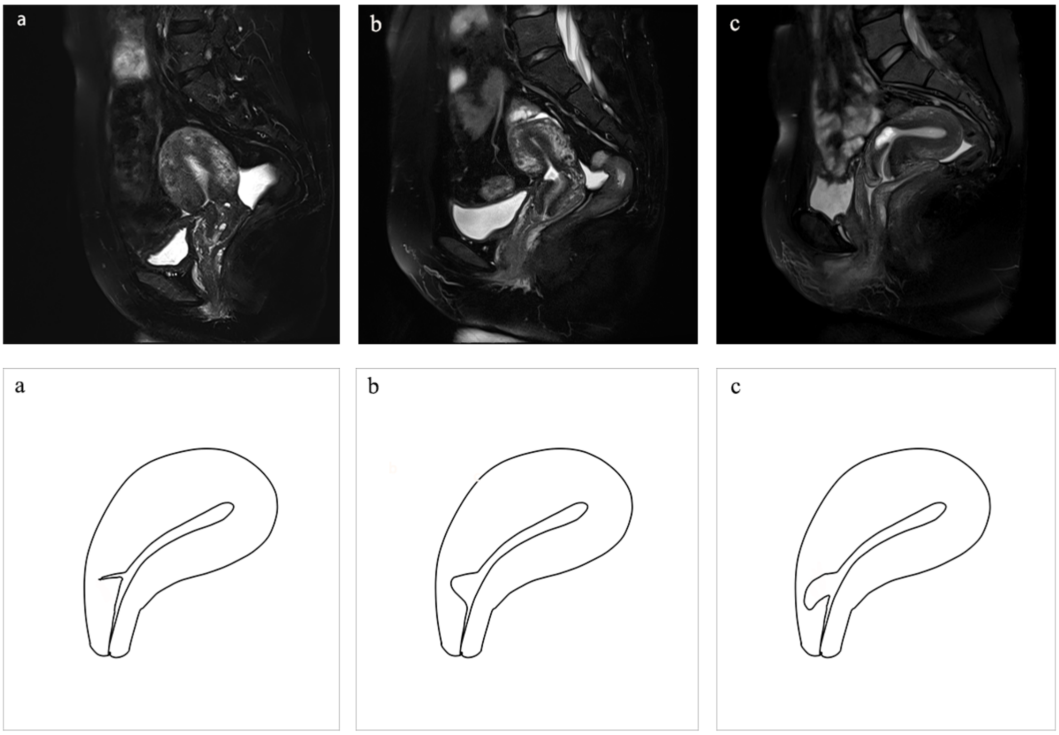

Measuring a niche in the sagittal plane

The shapes of niche are classification: (a) linear; (b) triangular; (c) irregular.

Figure S4. Various angles of niche.

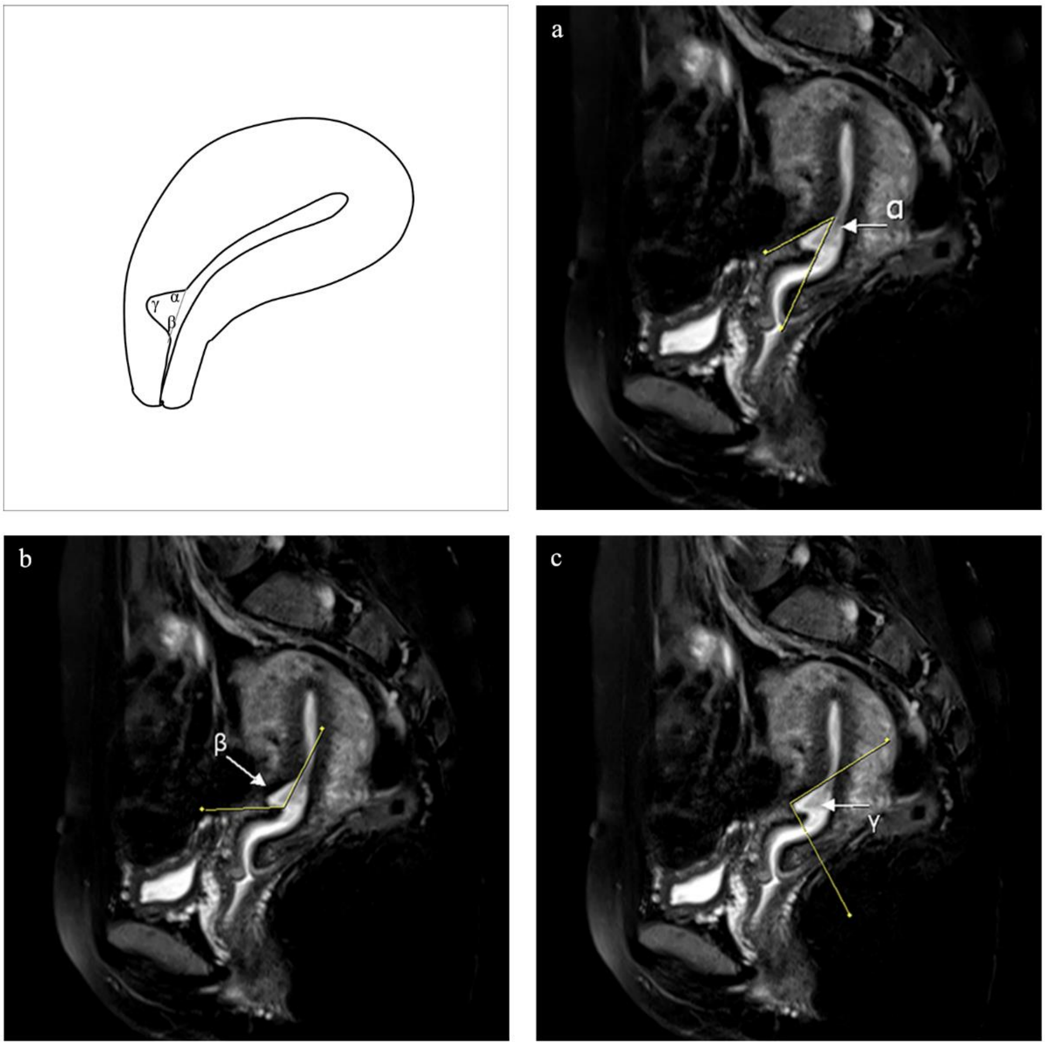

The angle of niche's endpoint at magnetic resonance imaging in the sagittal plane.

α: the angle of upper margin; β: the angle of lower margin; γ: the angle of apex;

Figure S5. Niche's endpoint measurement.

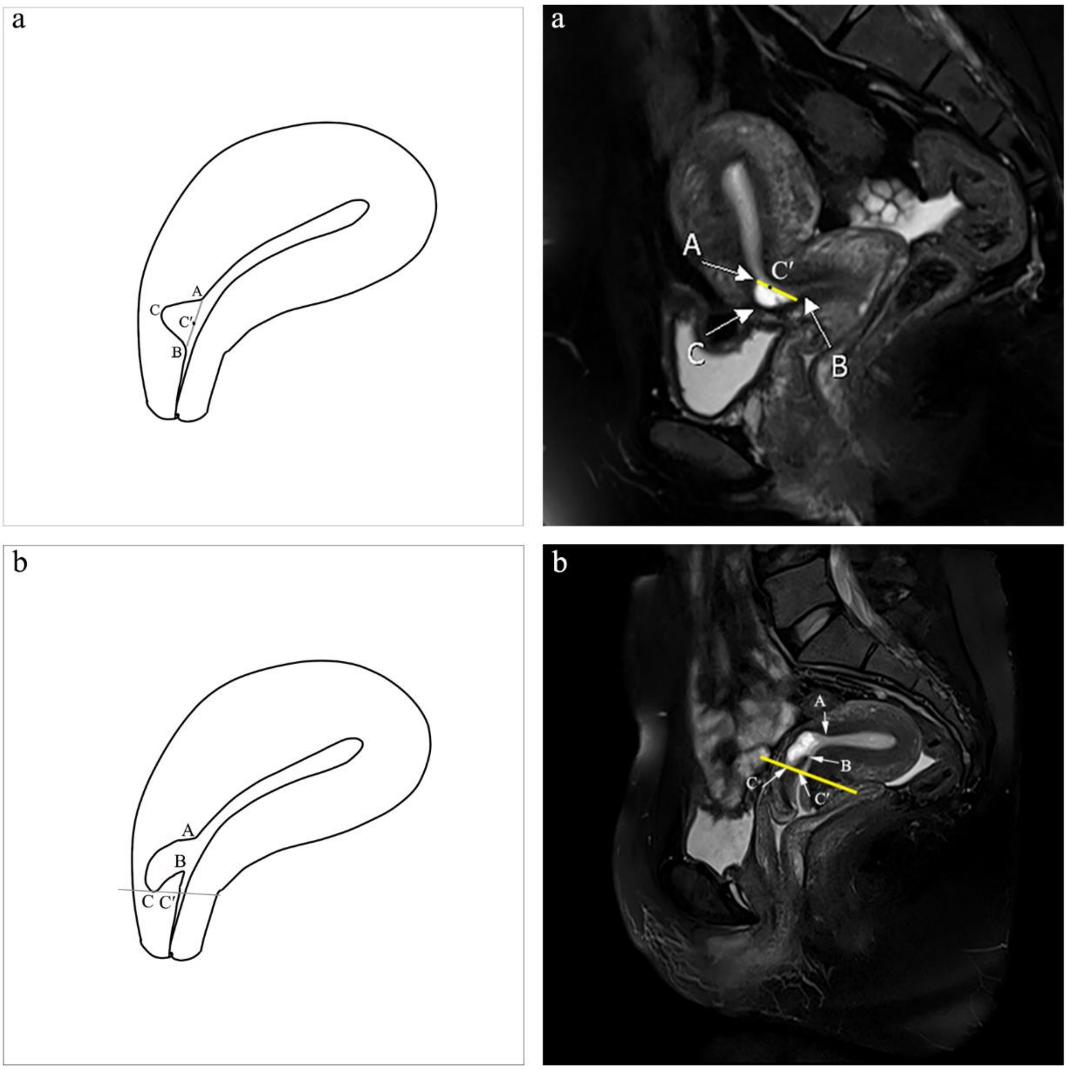

Niche's endpoint measurement at magnetic resonance imaging in the sagittal plane.

A: the endpoint of upper edge, B: the endpoint of lower edge; C: the apex of niche;

C′: the projection point of C on the line between A and B;

(a) C′ located within A and B; (b) C′ located outside the line between Aand B；

Figure S6. Schematic diagram and MRI of niche characteristics.

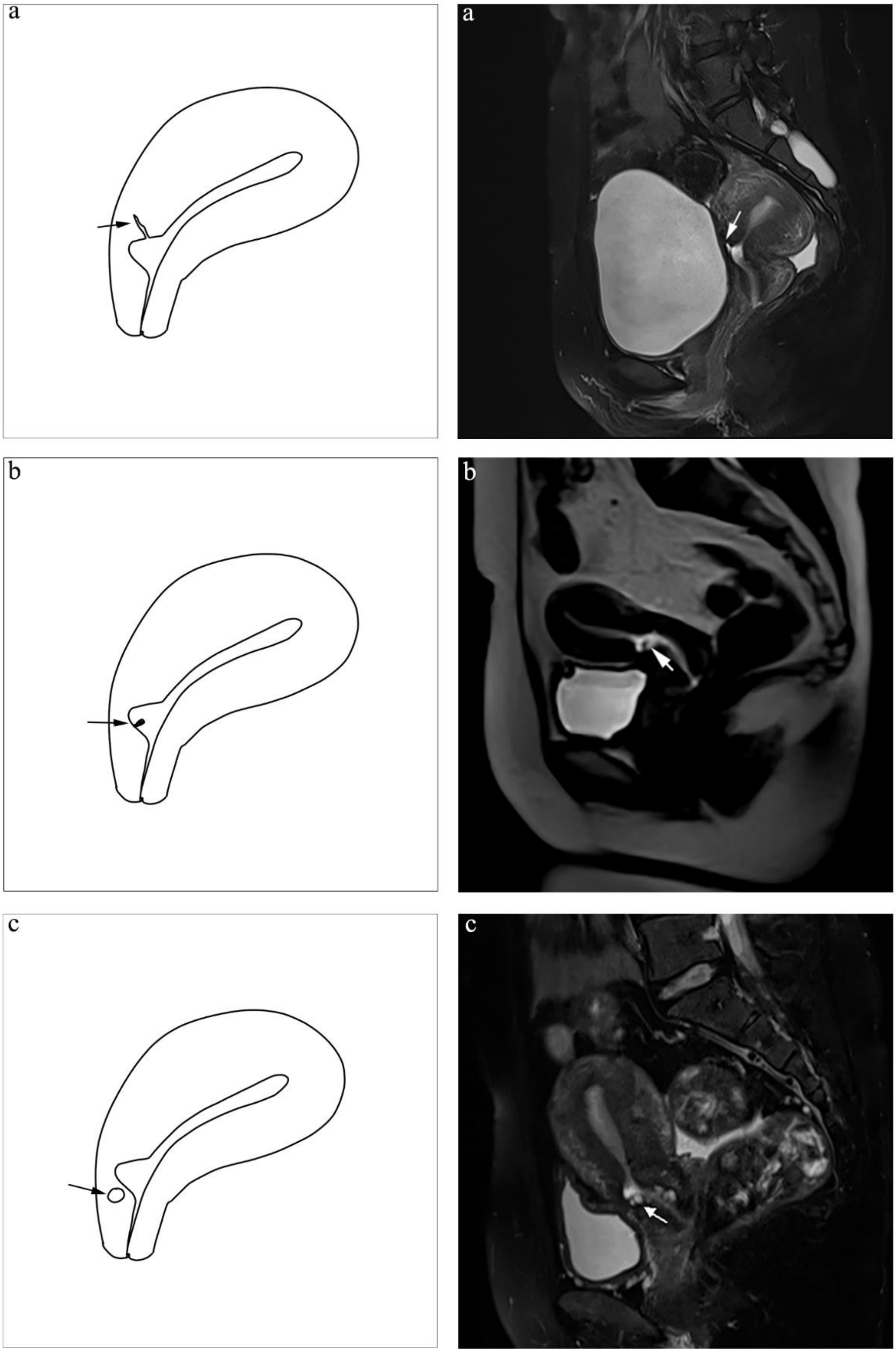

Schematic diagram and magnetic resonance imaging of niche characteristics: (a) lateral branch; (b) polyp-like structures; (c) cyst-like formations.

Figure S7. The position of the corpus in relation to the cervix.

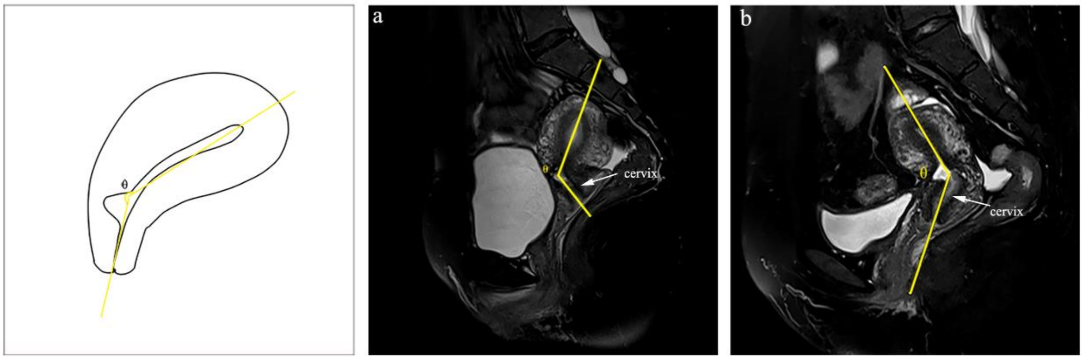

Figure S8. The measurement of width of the niche in the coronal plane.

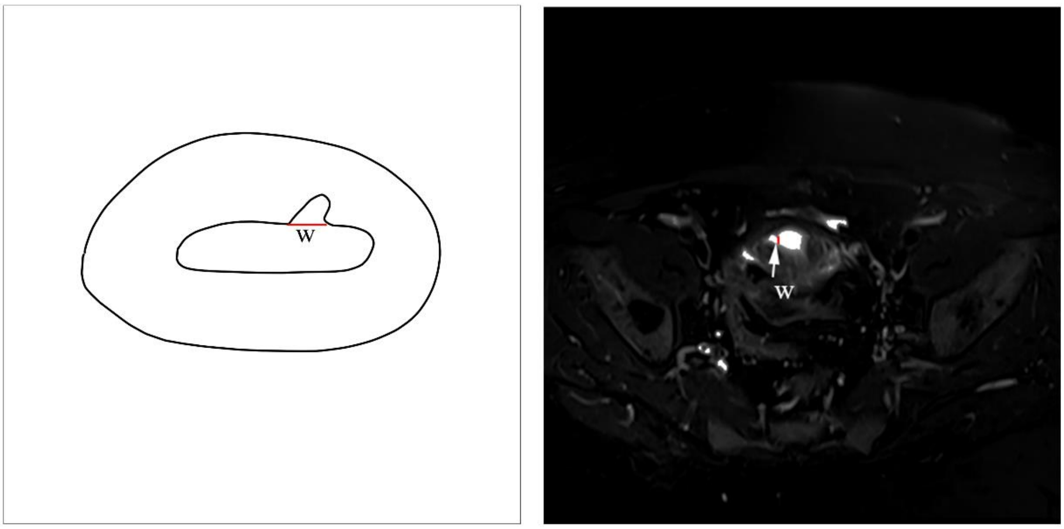

Measuring a niche in the coronal plane

Width is defined as the distance of the base of the defect in the coronal plane.

Appendix S1

MRI image acquisition and measurement procedure of niche.

Magnetic resonance imaging (MRI) was done on a 1.5 Tesla (Siemens Avanto, Erlangen, Germany) system with an actively shielded whole-body superconducting magnet. All the MRI were performed before the surgery, consist of T1-weighted (T1W) and T2-weighted (T2W) imaging sequences in sagittal and coronal planes of niche. Niche was diagnosed if a high signal intensity mass was seen in the normal myometrium of the anterior wall of lower segment uterus in the T1-weighted and T2-weighted images. Imaging measurement was performed by 2 observers who were blind to clinical effect and prior MRI reports with the Siemens Syngo MR software package.

The image measurement procedure

All measurements were conducted in sagittal and coronal planes. The length, depth and area of the niche should each be measured in the sagittal plane in which it is largest. TRM should be measured in the sagittal plane in which the niche has the smallest TRM. Width was measured in the coronal plane in which it is largest.

(1) Length (l) was measured from the upper rim to the lower rim of niche (Figure S1-a1);

(2) Depth (d) was defined as the vertical distance between the base and the apex of the defect (Figure S1-a2);

(3) TRM was defined as the distance from the serosal surface of the uterus to the apex of the niche (Figure S1-a1);

(4) TAM was defined as the total thickness adjacent to the niche next to the base of the defect (Figure S1-a1);

(5) The degree of severity of the defect was based on the ratio of the myometrial thickness at the scar to the thickness of adjacent myometrium. Severe defect was a ratio of <50% and mild defect was a ratio of ≥50%;

(6) The distance between the lower point of niche to the internal cervical os (d1, Figure S1-a2);

(7) The distance between the lowest demarcation of niche to the internal cervical os (d2, Figure S1-a2).

(8) Area was defined as the max area in the sagittal plane (Figure S2);

(9) The shapes of MRI diagnosed niche are (a) linear, (b) triangular, and (c) irregular rectangular in shape (Figure S3)

(10) Angle α was defined as the angle of the lower margin of the defect (Figure S4-a);

(11) Angle β was defined as the angle of upper margin of the defect (Figure S4-b);

(12) Angle γ was defined as the angle of the apex of the defect (Figure S4-c);

(13) The projection point of the apex of niche (C) on the line between the endpoint of upper edge (A) and the endpoint of lower edge (B) of niche: (a)Within A and B (Figure 5-a), (b) Outside A and B (Figure 5-b)

(14) The lateral branch was defined as the branch of niche, of which the widest part<2mm (Figure S6-a);

(15) The polyp-like structures were diagnosed as circular high signal intensity structure near the cervix in the T1-weighted and T2-weighted images (Figure S6-b);

(16) The cyst-like formations were defined as a low to intermediate signal intensity mass was seen in the niche's cavity in the T1-weighted and T2-weighted images (Figure S6-c);

(17) Angle θ was defined as angle between the cervical axis and the axis of uterine corpus ((Figure S7);

(18) Width was defined as the distance of the base of the defect in the coronal plane (Figure S8).

Table S1

Table S1. Characteristics of 50 patients undergoing hysteroscopic niche resection

Variables	(n = 50)
**Age (years)**	34.40±4.26
**Gravidity**	2 (1,4)
**Parity**	1.42±0.61
**Number of caesarean sections**	1 (1, 2)
**Preoperative menstrual duration (days)**	13 (10, 14)
**Preoperative dysmenorrhea score (0-10)**	0 (0, 3)
**Preoperative chronic pelvic pain score (0-10)**	0 (0, 3)
**Which symptom improvement is most important to you**	
postmenstrual spotting	45 (90%)
dysmenorrhea	3 (6%)
chronic pelvic pain	2 (4%)
**The minimal days of shortening in postmenstrual spotting duration to be satisfied (day).**	
**⩾2**	5 (10%)
⩾3	37 (74%)
⩾4	6 (12%)
⩾5	2 (4%)

Data are reported as mean ± standard deviation or median (interquartile range, IQR) or as n (valid percentage)

Table S2

Table S2. Preoperative MRI findings for all participants

Variables	All participants (n = 208)
length (mm)	8.14±3.49
depth (mm)	5.51±1.85
TRM (mm)	3.41±1.53
TAM (mm)	9.78±2.25
The degree of severity of the defect (TRM/TAM)	
Angle (α)	65.50±19.41
Angle (β)	78.85±29.61
Angle (γ)	100.08±32.33
Angle (θ)	193.56±63.34
Area (mm^2)	28.63±10.19
Lateral branch	
YES	22 (10.58%)
NO	186 (89.42%)
Shape	
Linear	20 (9.62%)
Triangle	42 (20.19%)
Irregular rectangular	146 (70.19%)
The projection point of the C on the line between A and B	
Within A and B	165 (79.33%)
Outside A and B	43 (20.67%)
The distance between the lower point of niche to the internal cervical os (mm)	2.75±1.72
The distance between the lowest demarcation of niche to the internal cervical os (mm)	6.09±2.41
Polyp in the cavity of niche	
YES	14 (6.73%)
NO	194 (93.27%)
Cyst in the cavity of niche	
YES	17 (8.17%)
NO	191 (91.83%)

MRI=magnetic resonance image; TRM=thickness of the residual myometrium; TAM=thickness of the adjacent myometrium; TRM/TAM= the ratio of myometrial thickness at the scar to the thickness of adjacent myometrium

Angle α was defined as the angle of the upper margin of the defect; Angle β was defined as the angle of lower margin of the defect; Angle γ was defined as the angle of the apex of the defect; Angle θ was defined as angle between the cervical axis and the axis of uterine corpus
A: the endpoint of upper edge of niche on MRI; B: the endpoint of lower edge of niche on MRI; C: the apex of niche on MRI

Data are reported as mean ± standard deviation as n (valid percentage)
